# Patterns of dendritic cell and monocyte subsets are associated with disease severity and mortality in liver cirrhosis patients

**DOI:** 10.1038/s41598-021-85148-y

**Published:** 2021-03-15

**Authors:** Chandra Chiappin Cardoso, Camila Matiollo, Carolina Hilgert Jacobsen Pereira, Janaina Santana Fonseca, Helder Emmanuel Leite Alves, Otavio Marcos da Silva, Vivian de Souza Menegassi, Claudia Regina dos Santos, Ana Carolina Rabello de Moraes, Leonardo de Lucca Schiavon, Maria Claudia Santos-Silva

**Affiliations:** 1 Division of Clinical Analysis, Flow Cytometry Service, Health Sciences Center, University Hospital of the Federal University of Santa Catarina, Florianópolis, SC 88040-900 Brazil; 2 Postgraduate Program in Pharmacy of the Federal University of Santa Catarina, Florianópolis, SC Brazil; 3 Postgraduate Program in Medical Sciences of the Federal University of Santa Catarina, Florianópolis, SC Brazil; 4 Division of Gastroenterology, Federal University of Santa Catarina, Florianópolis, SC Brazil; 5 Clinical Analysis Department, Health Sciences Center, Federal University of Santa Catarina, Florianópolis, SC Brazil

**Keywords:** Conventional dendritic cells, Plasmacytoid dendritic cells, Monocytes and macrophages, Immunological deficiency syndromes

## Abstract

Liver cirrhosis is often complicated by an immunological imbalance known as cirrhosis-associated immune dysfunction. This study aimed to investigate disturbances in circulating monocytes and dendritic cells in patients with acute decompensation (AD) of cirrhosis. The sample included 39 adult cirrhotic patients hospitalized for AD, 29 patients with stable cirrhosis (SC), and 30 healthy controls (CTR). Flow cytometry was used to analyze monocyte and dendritic cell subsets in whole blood and quantify cytokines in plasma samples. Cirrhotic groups showed higher frequencies of intermediate monocytes (iMo) than CTR. AD patients had lower percentages of nonclassical monocytes than CTR and SC. Cirrhotic patients had a profound reduction in absolute and relative dendritic cell numbers compared with CTR and showed higher plasmacytoid/classical dendritic cell ratios. Increased plasma levels of IL-6, IL-10, and IL-17A, elevated percentages of CD62L^+^ monocytes, and reduced HLA-DR expression on classical monocytes (cMo) were also observed in cirrhotic patients. Patients with more advanced liver disease showed increased cMo and reduced tissue macrophages (TiMas) frequencies. It was found that cMo percentages greater than 90.0% within the monocyte compartment and iMo and TiMas percentages lower than 5.7% and 8.6%, respectively, were associated with increased 90-day mortality. Monocytes and dendritic cells are deeply altered in cirrhotic patients, and subset profiles differ between stable and advanced liver disease. High cMo and low TiMas frequencies may be useful biomarkers of disease severity and mortality in liver cirrhosis.

## Introduction

Monocytes, macrophages, and dendritic cells (DCs) are mononuclear phagocytes expressing major histocompatibility complex (MHC) class II molecules. These efficient antigen-presenting cells can be found in virtually all tissues. Circulating monocytes can be divided into three subtypes according to their function and phenotype. Classical monocytes (cMo) are CD14^++^ CD16^−^, intermediate monocytes (iMo) are CD14^+^ CD16^+^, and nonclassical monocytes (ncMo) are CD14^−/low^ CD16^+^^1, 2^. iMo and ncMo are also classified as tissue macrophages (TiMas). It is known that these subsets originate from the cMo lineage, but it is still unclear whether differentiation occurs in circulation or body tissues^3–5^. iMo, characterized by positive expression of CCR5 and intermediate expression of CCR2 and CX3CR1, was the last of the three subsets to be identified. Moreover, a recent study proposed the existence of novel monocyte subsets, defined by expression patterns of chemokine receptors, FcR, and adhesion molecules^6^.

DCs have several immunological functions. They detect homeostatic imbalances, secrete cytokines and growth factors, and process antigens for presentation to T helper (Th) cells, inducing naïve T-cell activation and effector differentiation. Additionally, DCs can present extracellular antigens via MHC-I to cytotoxic T cells (Tc)^2,7–9^. In blood, DCs can be broadly divided into two subtypes: myeloid or classical DCs (cDCs) and plasmacytoid DCs (pDCs)^9,10^. Phenotypically, cDCs are identified as CD11c^++^ CD123^dim^ and can be further divided into two subsets, cDC1 (CD141^+^) and cDC2 (CD1c^+^). cDC1 cells play crucial roles in antiviral and antitumoral immunity by presenting extracellular antigens via MHC-I to Tc. cDC2, in contrast, present antigens to Th cells^7–9^. Activation of CD4^+^ T cells mediated by cDCs results in their phenotypic polarization to proinflammatory Th1 and Th17, anti-inflammatory Th2, or immunoregulatory Treg cells^11^. pDCs, defined as CD11c^dim^ CD123^++^, are extremely important in protecting against viral infection, as they secrete high amounts of type I interferon (IFN-α/β) upon stimulation of toll-like receptors (TLRs) 7 and 9^12^. Moreover, pDCs seem to be implicated in defense responses to *Aspergillus fumigatus*, autoimmune diseases, chronic inflammation, and immune tolerance^9^. As compared with the cDC subtype, pDCs express high levels of IL-3Rα (CD123) and a different set of TLRs and are less effective in inducing T-cell activation^10,13^.

Patients with cirrhosis may develop a significant immunological imbalance, known as cirrhosis-associated immune dysfunction (CAID), whose characteristics vary according to the stage of the disease. Early cirrhosis is marked by exacerbated inflammation. In more advanced stages, the immune system collapses and patients suffer from immunodeficiency^14,15^. During the immunodeficiency phase, patients are vulnerable to bacterial infections and other serious complications that considerably increase morbidity and mortality^14,16,17^.

Previous studies reported monocyte disturbances in cirrhotic patients, including alterations in CD16^+^ subset and presence of unfunctional monocytes^18,19^. There is also evidence that circulating DCs are decreased in viral diseases, such as dengue infection^20^, hepatitis C virus (HCV) infection^21,22^, and chronic hepatitis B virus (HBV) infection^23^. Furthermore, disturbances in liver DC populations were observed in liver fibrosis^24^ and hepatocellular carcinoma^25^. However, data on circulating DCs and monocytes in cirrhosis and their relationship with disease severity and mortality are still scarce. In this study, we examined frequencies and absolute numbers of peripheral subsets of monocytes and DCs in healthy subjects and adult patients at different clinical stages of liver cirrhosis in order to determine whether these cells can be used as biomarkers of disease severity.

## Results

### Patients characteristics

This study included 98 subjects: 39 patients hospitalized for AD, 29 outpatients with SC, and 30 healthy subjects. CTR individuals, 66% of whom were men, were aged 50.90 ± 10.25 years. Demographic, laboratory, and clinical characteristics of SC and AD patients are described in Table 1. Alcohol consumption, HCV infection, and nonalcoholic steatohepatitis were the most common etiological factors. Expected differences were found between laboratory parameters of SC and AD individuals. The AD group had profound lymphopenia and anemia, higher serum levels of creatinine, bilirubin, and lactate, greater international normalized ratio value, and lower serum albumin levels. Spontaneous bacterial peritonitis was detected in four (10.3%) AD patients. Clinical features of patients were also compared. Most SC patients (89.7%) were classified as Child–Pugh A, whereas the same percentage of AD patients were classified as Child–Pugh B and C. The median MELD score of SC patients was 9, and that of AD patients was 16. Eight AD patients (20.5%) were diagnosed with ACLF (seven with ACLF-1 and one with ACLF-2). Of all SC patients, eight (27.6%) had never been hospitalized for the disease or presented with clinical complications, such as ascites, hepatic encephalopathy (HE), and gastrointestinal bleeding; these patients were considered to have compensated cirrhosis (CC).

**Table 1:** Characteristic of the cirrhotic patients.

Variable	Stable cirrhosis (n = 29)	Acute decompensation (n = 39)	*p*
Age (years), mean ± SD	58.72 ± 7.49	56.31 ± 11.59	0.301
Male gender, n (%)	23 (79.3)	27 (69.2)	0.351
Diabetes mellitus, n (%)	11 (37.9)	15 (38.5)	0.964
**Etiology of cirrhosis, n (%)**			
Alcohol	15 (51.7)	15 (38.5)	0.309
Hepatitis C	2 (6.9)	8 (20.5)	0.232
Hepatitis B	1 (3.5)	1 (2.6)	0.611
Alcohol and viral	2 (6.9)	3 (7.7)	0.640
NASH	3 (10.3)	7 (17.9)	0.498
Cryptogenic	2 (6.9)	3 (7.7)	0.640
Others^†^	4 (13.8)	2 (5.1)	0.390
**Laboratorial and clinical features**			
Leukocyte/mm^3^, median (range)	6100 (2620–10,530)	5120 (1010–14,510)	0.687
Lymphocytes/mm^3^, median (range)	1328 (480–3970)	840 (161–2250)	0.001
RBC million/mm^3^, mean ± SD	4.57 ± 0.49	3.24 ± 0.65	< 0.001
Hemoglobin (g/dL), median (range)	14.6 (7.6–17.5)	9.5 (6.3–15.0)	< 0.001
Hematocrit (%), median (range)	42.7 (27.8–51.9)	28.3 (20.0–43.3)	< 0.001
Platelets/mm^3^, median (range)	104,000 (37,000–234,000)	68,000 (18,000–259,000)	0.070
Sodium (mEq/L), mean ± SD	138.4 ± 3.0	136.6 ± 5.3	0.086
Creatinine (mg/dL), median (range)	0.88 (0.62–2.08)	1.15 (0.55–6.19)	0.001
INR, median (range)	1.11 (0.96–1.64)	1.44 (1.04–2.58)	< 0.001
Albumin (g/dL), median (range)	3.81 ± 0.42	2.88 ± 0.68	< 0.001
CRP (mg/L), median (range)	3.20 (0.50–22.70)	21.90 (3.2–172.90)	< 0.001
Total bilirubin (mg/dL), median (range)	1.00 (0.30–5.50)	2.10 (0.20–8.90)	0.006
Lactate (mmol/L), median (range)	1.20 (0.40–3.20)	1.65 (0.80–4.90)	0.003
AST (IU/L), median (range)	33.00 (16.00–143.00)	46.00 (15.00–260.00)	0.052
ALT (IU/L), median (range)	34.00 (16.00–133.00)	32.00 (8.00–165.00)	0.378
GGT (IU/L), median (range)	80.50 (30.00–1165.00)	107.00 (18.00–536.00)	0.391
Child–Pugh, median (range)	5 (5–8)	9 (6–14)	< 0.001
Child–Pugh A, n (%)	26 (89.7)	4 (10.3)	< 0.001
Child–Pugh B, n (%)	3 (10.3)	19 (48.7)	0.001
Child–Pugh C, n (%)	0 (0.0)	16 (41.0)	< 0.001
MELD, median (range)	9 (7–19)	16 (8–31)	< 0.001
MELD < 9, n (%)	19 (65.5)	3 (7.7)	< 0.001
MELD 10–19, n (%)	10 (34.5)	27 (69.2)	0.004
MELD 20–29, n (%)	0 (0.0)	9 (23.1)	0.008
Ascites, n (%)	0 (0.0)	26 (66.7)	< 0.001
Grade I, n (%)	0 (0.0)	12 (30.8)	0.001
Grade II e III, n (%)	0 (0.0)	14 (35.9)	< 0.001
HE, n (%)	0 (0.0)	19 (48.7)	< 0.001
Grade I, n (%)	0 (0.0)	8 (20.5)	0.017
Grade II e III, n (%)	0 (0.0)	11 (28.2)	0.002
Gastrointestinal bleeding, n (%)	0 (0.0)	17 (43.6)	< 0.001
ACLF, n (%)	–	8 (20.5)	–
Grade 1, n (%)	–	7 (17.9)	–
Grade 2, n (%)	–	1 (2.6)	–
SBP, n (%)	–	4 (10.3)	–

*AST* Aspartate aminotransferase, *ALT* Alanine aminotransferase, *CRP* C-reactive protein, *GGT* Gamma-glutamyl transferase, *HE* Hepatic encephalopathy, *INR* International normalized ratio, *MELD* Model for end-stage liver disease, *RBC* Red blood cells, *SPB* Spontaneous bacterial peritonitis. *NASH* Nonalcoholic steatohepatitis.

^†^Other etiologic factors included hemochromatosis, Wilson's disease, and secondary biliary cirrhosis.

### Monocyte and DC subsets are altered in liver cirrhosis patients compared with healthy controls

**Figure 1:**
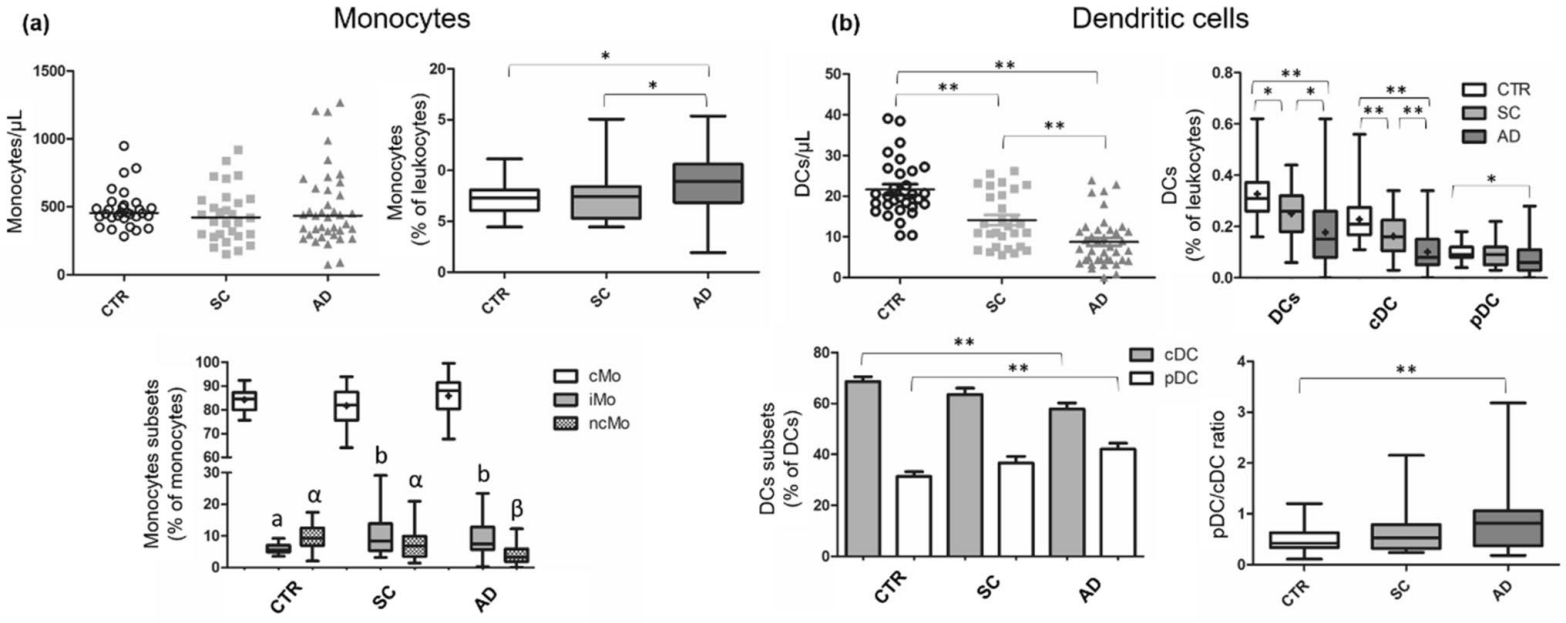
Absolute and relative numbers of monocytes and DCs in healthy controls, stable cirrhosis and in acute decompensation of cirrhosis. A series of comparisons of absolute and relative numbers of monocytes (Panel **a**) and DCs (Panel **b**) obtained by manual gating and differences among study groups. *p ≤ 0.05; **p ≤ 0.01. In Panel (**a**), a and b: difference between SC and CTR and AD and CTR (*p* ≤ 0.01), α and β: difference between AD and CTR and AD and SC (*p* ≤ 0.01). *AD* Acute decompensation of cirrhosis, *cDC* Classical dendritic cell, *cMo* Classical monocytes, *CTR* Healthy controls, *DCs* Dendritic cells, *iMo* Intermediate monocytes, *ncMo* Nonclassical monocytes, *pDC* Plasmacytoid dendritic cell, *SC* Stable cirrhosis. Differences were tested using Kruskal–Wallis or ANOVA/Bonferroni. Parametric variables are represented by boxplot with a “ + ” (mean) or by column chart.

Cirrhotic patients had a higher percentage of monocytes than CTR, but this difference was not apparent in absolute numbers (Fig. 1a). Significant differences were observed in monocyte subset (cMo, iMo, and ncMo) frequencies. As expected, cMo was the predominant subset in CTR patients, followed by ncMo and iMo. Both cirrhotic groups had higher frequencies of iMo (CD14^+^CD16^+^) than CTR. The AD group had a lower frequency of ncMo than CTR and SC (*p* ≤ 0.01). No significant differences in cMo percentages within the monocyte compartment were observed between groups. However, when evaluating the proportion of cMo in relation to leukocytes, we found that AD patients had higher cMo percentages than CTR and SC individuals (*p* ≤ 0.05). Cirrhotic patients, particularly those with AD, showed a substantial reduction in absolute and relative numbers of circulating DCs compared with CTR. Significant differences were found between SC and AD groups, as depicted in Fig. 1b. Interestingly, when considering only the DC compartment, we observed that AD patients showed reduced cDC and increased pDC percentages, resulting in an increased pDC/cDC ratio. Table S2 (Supplementary material) shows monocyte and DC subsets frequencies of the three groups evaluated in this study.

**Figure 2:**
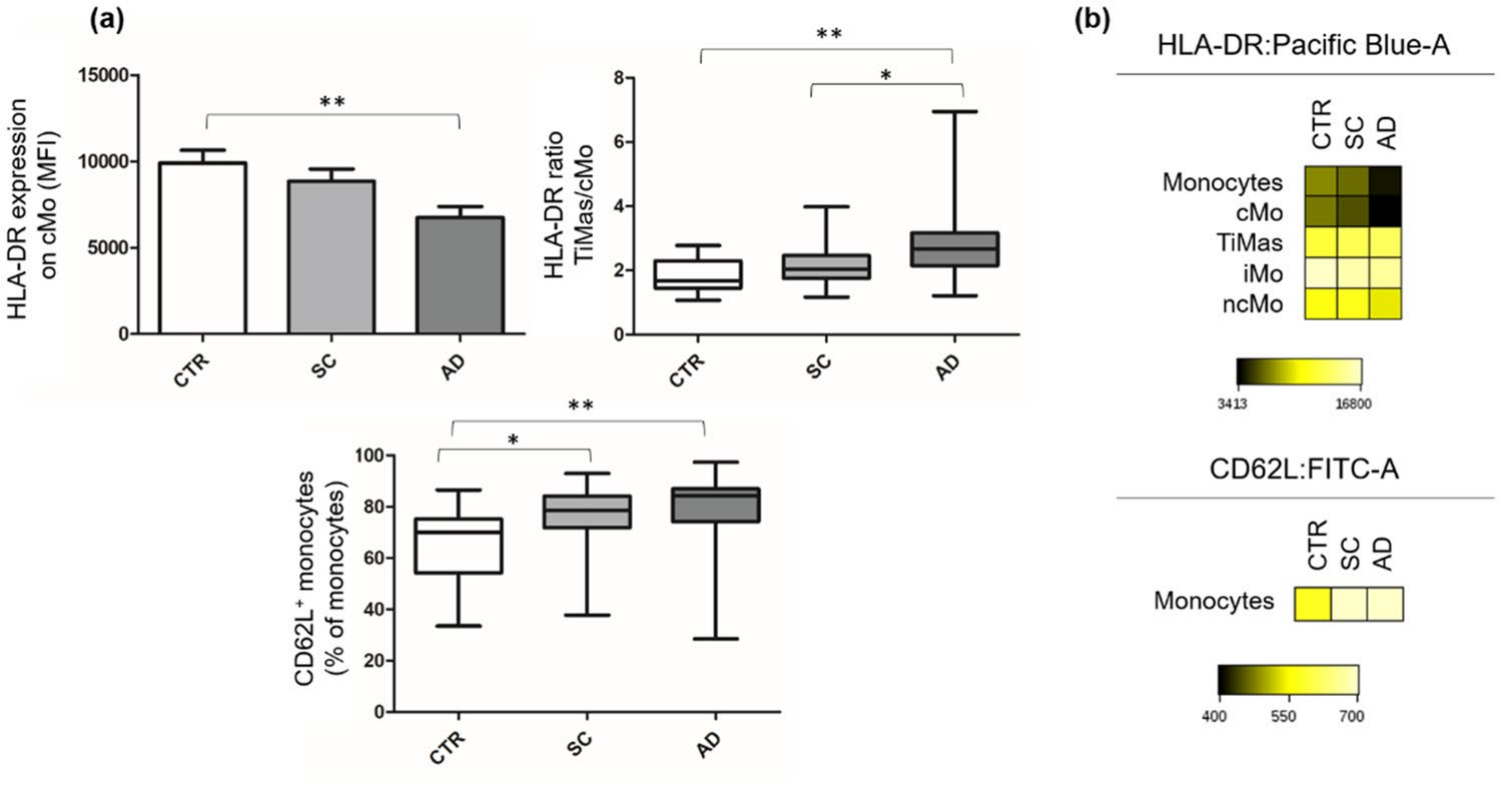
HLA-DR and CD62L expression on monocytes. Panel (**a**) HLA-DR and CD62L expression on monocytes of healthy controls, stable cirrhosis and in acute decompensation of cirrhosis.Values are represented as MFI of HLA-DR expression and percentage of CD62L^+^ monocytes. Panel (**b**) Heatmap of the FCS files of 30 CTR, 29 SC and 39 AD merged together. The colors in the heatmap represent the median of the intensity of HLA-DR and CD62L expression on monocytes, varying from black for lower expression to light yellow for higher expression. *p ≤ 0.05; **p ≤ 0.01. *AD* Acute decompensation of cirrhosis, *cMo* Classical monocytes, *CTR* Healthy controls, *MFI* Mean fluorescence intensity, *SC* Stable cirrhosis, *TiMas* Tissular macrophages. Differences were tested using Kruskal–Wallis or ANOVA/Bonferroni. Parametric variables are represented by column chart.

Monocyte surface expression of HLA-DR was investigated. For this, we measured the MFI of HLA-DR on cMo, iMo, ncMo, and TiMas and calculated the TiMas/cMo HLA-DR ratio. No differences in iMo, ncMo, or TiMas HLA-DR expression were observed between groups. However, AD patients showed a lower MFI of cMo HLA-DR than CTR individuals (*p* ≤ 0.01) and a higher TiMas/cMo HLA-DR ratio than the other groups (Fig. 2 a,b). Monocyte expression of the adhesion molecule CD62L was also assessed. SC and AD showed higher percentages of CD62L^+^ monocytes than CTR (*p* ≤ 0.05 and *p* ≤ 0.01, respectively) (Fig. 2 a,b).

### Cytokine measurements in liver cirrhosis patients

**Figure 3:**
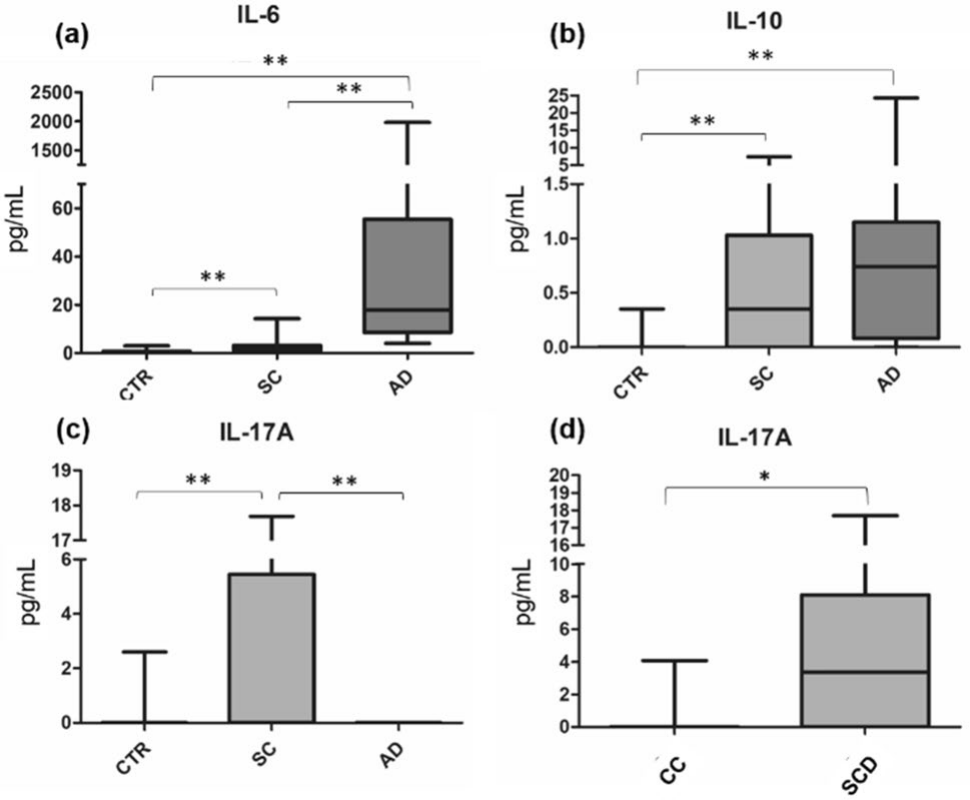
IL-6, IL-10 and IL-17A plasma levels in healthy controls, stable cirrhosis and in acute decompensation of cirrhosis. Plasma levels of IL-6 (**a**), IL-10 (**b**) and IL-17A (**c**) in healthy controls, stable cirrhosis and in acute decompensation of cirrhosis. Panel d: comparison between patients with compensated cirrhosis (CC) and patients with stable cirrhosis that had already developed decompensated cirrhosis (SCD). *p ≤ 0.05; **p ≤ 0.01. *AD* Acute decompensation of cirrhosis, *CTR* Healthy controls, *IL* Interleukine. Differences were tested using Kruskal–Wallis.

Cytokine measurements demonstrated that IL-6 was very elevated in cirrhotic patients, particularly in AD individuals (Fig. 3a. Similar results were observed for IL-10 but without differences between cirrhotic groups (Fig. 3b). IL-17A was found to be elevated in SC patients (Fig. 3 c,d). Noteworthy, this cytokine was high in SC patients with previous clinical complications or hospitalization for liver disease (SCD) in comparison with SC patients with compensated cirrhosis (CC) (*p* ≤ 0.05). In fact, this was the only parameter that differed significantly when the SC group was compared according to these features.

### Monocyte and DC alterations are associated with cirrhosis progression and clinical complications

Spearman’s correlation coefficient was used to identify correlations between numerical variables of interest. DC and monocyte frequencies were compared according to MELD and Child–Pugh scores (Table S3, Supplementary material). The results demonstrated that patients with more advanced disease have higher cMo frequencies, lower TiMas frequencies, and lower DC frequencies. A moderate positive correlation was found between TiMas/cMo HLA-DR ratio and MELD score. Regarding the DC compartment, high pDC frequency and pDC/cDC ratio correlated moderately and positively with Child–Pugh score. IL-6 correlated positively with MELD and Child–Pugh scores, whereas IL-17A correlated negatively with the scores.

**Figure 4:**
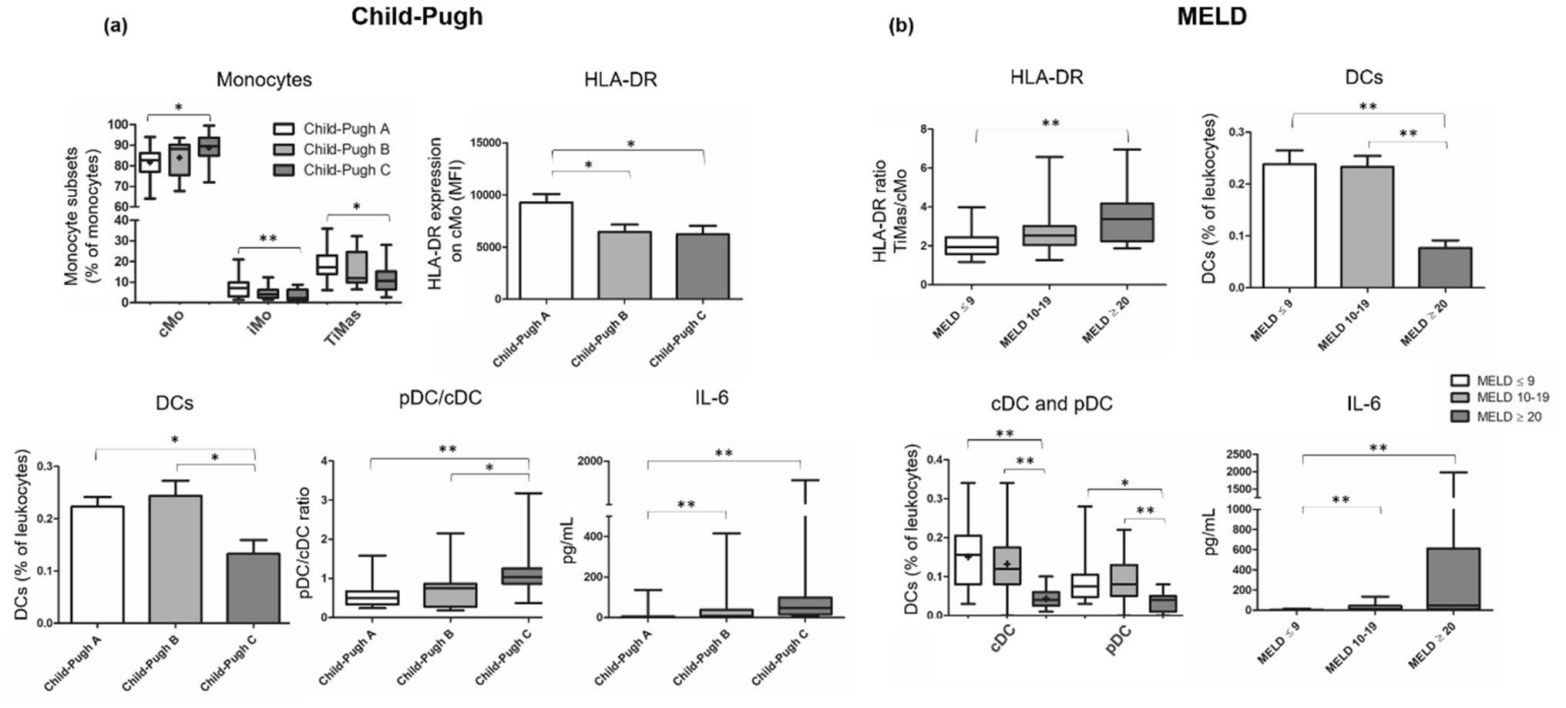
Monocytes, DCs and IL-6 in cirrhotic patients classifed according to Child–Pugh and MELD scores. A series of comparisons of relative numbers of monocytes and DCs and plasma levels of IL-6 of cirrhotic patients classified as Child–Pugh A, B or C (Panel a), and as MELD ≤ 9, 10–19 or ≥ 20 (Panel b). *p ≤ 0.05; **p ≤ 0.01. Differences were tested using Kruskal–Wallis or ANOVA/Bonferroni. Parametric variables are represented by boxplot with a “ + ” (mean) or by column charts.

MELD scores were categorized into three groups (≤ 9, 10–19, and ≥ 20) and Child–Pugh scores into three classes (A, B, and C). Patients with advanced liver disease showed altered distribution of monocyte subsets, lower HLA-DR expression on cMo, lower DC frequencies, and higher pDC proportion within the DC compartment (Fig. 4). Cirrhotic patients with advanced liver disease (MELD score of 20–29 or Child–Pugh C) had highly elevated IL-6 plasma levels compared with patients with initial or stable liver disease (MELD ≤ 9 and Child–Pugh A).

We also compared cirrhotic patients according to the presence of ascites and HE (Table 2). Ascitic patients showed higher cMo and lower TiMas frequencies within the monocyte compartment as well as lower DC frequencies. Similar results were observed for patients with HE.

**Table 2:** Monocytes and DCs in cirrhotic patients in relation to the presence of ascites and hepatic encephalopathy.

	Cirrhosis without ascites (n = 42)	Cirrhosis with ascites (n = 26)	*p*
Monocytes %, median (range)	7.38 (1.92–16.57)	8.51 (3.16–16.70)	0.065
cMo %, mean ± SD	6.35 ± 2.61	8.19 ± 3.13	0.011
cMo^†^, mean ± SD	81.84 ± 8.05	87.69 ± 7.13	0.003
TiMas^†^, mean ± SD	18.17 ± 8.05	12.43 ± 6.94	0.004
DCs %, mean ± SD	0.25 ± 0.12	0.15 ± 0.10	0.001
cDC %, mean ± SD	0.16 ± 0.08	0.08 ± 0.06	< 0.001
cDC^‡^, mean ± SD	63.12 ± 13.38	55.68 ± 14.55	0.033
pDC^‡^, mean ± SD	36.88 ± 13.18	44.29 ± 14.56	0.034

	Cirrhosis without HE (n = 49)	Cirrhosis with HE (n = 19)	*p*
Monocytes%, median (range)	7.60 (1.92–16.70)	8.71 (4.26–15.48)	0.100
cMo%, mean ± SD	6.60 ± 2.95	8.23 ± 2.65	0.039
cMo^†^, mean ± SD	82.26 ± 8.20	88.76 ± 6.11	0.001
iMo^†^, median (range)	8.49 (3.20–29.12)	6.57 (0.29–23.45)	0.034
NcMo%, median (range)	0.43 (0.04–1.66)	0.25 (0.01–0.94)	0.038
NcMo^†^, median (range)	5.72 (0.43–20.97)	3.70 (0.08–8.68)	0.009
TiMas^†^, mean ± SD	17.74 ± 8.20	11.40 ± 5.83	0.001
DCs%, mean ± SD	0.23 ± 0.12	0.16 ± 0.12	0.042
cDC%, mean ± SD	0.14 ± 0.08	0.09 ± 0.07	0.008
cDC mm^3^, median (range)	6 (1–18)	4 (0–13)	0.014
IL-10, median (range)	0.24 (0.00–7.46)	0.92 (0.00–24.25)	0.001
IL-6, median (range)	4.06 (0.34–800.95)	32.48 (4.36–1978.06)	< 0.001

*cDC* Classical dendritic cell, *cMo* Classical monocytes, *DCs* Dendritic cells, *HE* Hepatic encephalopathy, *IL* Interleukine, *iMo* Intermediate monocytes, *ncMo* Nonclassical monocytes, *pDC* Plasmacytoid dendritic cell, *TiMas* Tissular macrophages.

^†^The percentage of monocytes subsets referred to as total monocytes.

^‡^The percentage of cDC and pDC referred to as total DCs. Statistical differences between groups were calculated using Mann–Whitney U and Student’s T-test tests.

Factors associated with ACLF are shown in Table S4 (Supplementary material). ACLF was associated with serum creatinine, C-reactive protein, alanine aminotransferase, IL-10, HE, MELD score, and iMo and DC percentages.

### Association between monocyte percentages and mortality

Six patients (15%) died within the first 90 days, all of them belonging to the AD group. Univariate Cox regression analysis was performed to investigate factors associated with 90-day mortality (Table S5, Supplementary material). Bacterial infection, Child–Pugh C classification, serum C-reactive protein, IL-6, percentages of cMo, iMo, and TiMas within the monocyte compartment, percentage of CD62L^+^ monocytes, and pDC/cDC ratio were associated with 90-day mortality. Multivariate Cox regression was not carried out because of the low number of events (six deaths).

**Figure 5:**
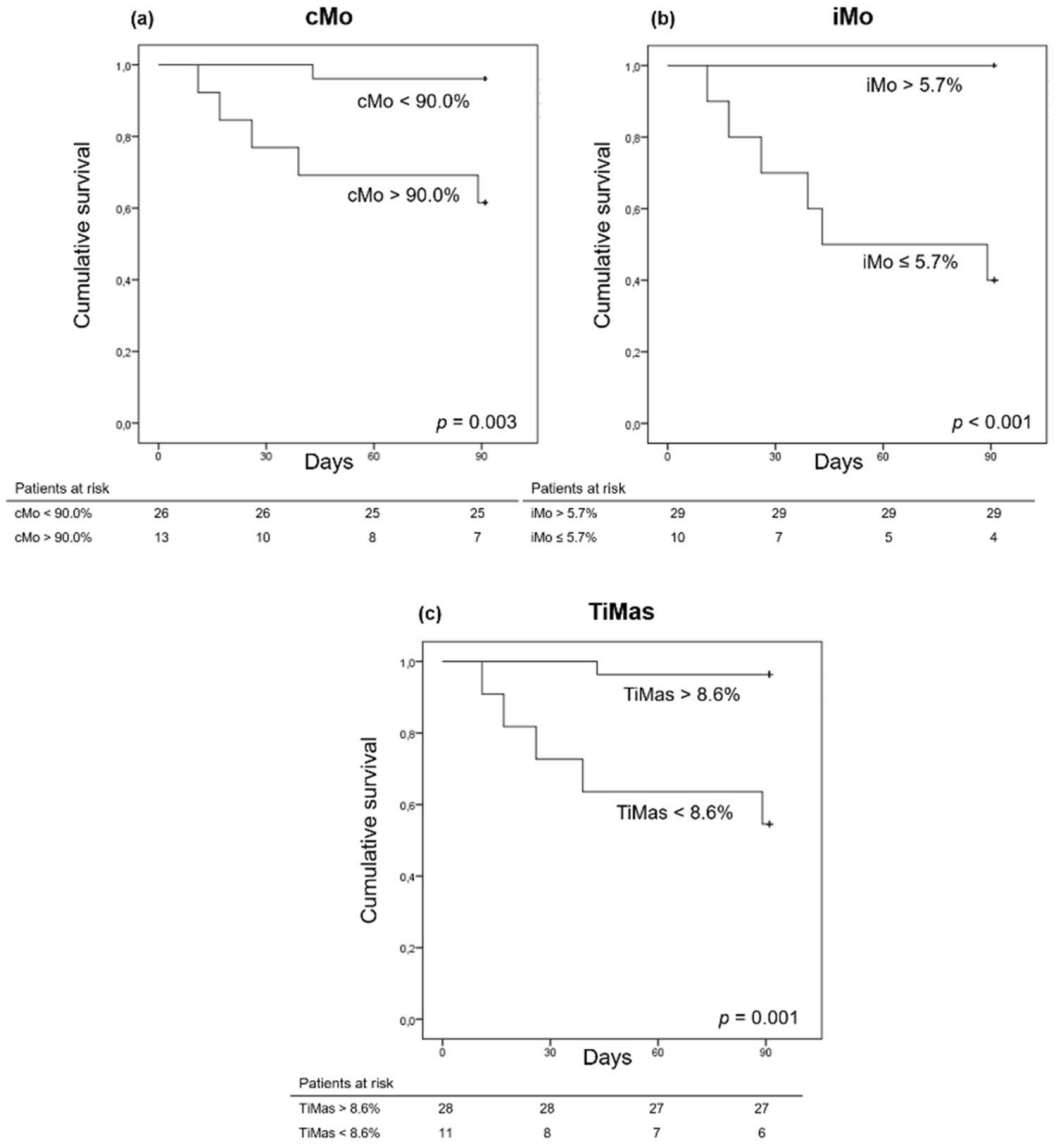
Cumulative 90-days survival of patients hospitalized with acute decompensation of cirrhosis according to monocyte subsets. The Kaplan–Meier survival probability was 96.2% in subjects with cMo frequencies < 90.0% and 61.5% in those with values > 90.0% (*p* = 0.003) (**a**), 100% in subjects with iMo frequencies > 5.7% and 60% in those with values ≤ 5.7% (*p* < 0.001) (**b**), and 96.4% in subjects with TiMas frequencies > 8.6% and 54.5% in those with values < 8.6% (*p* = 0.001) (**c**).

The AUROC of cMo percentage (within the monocyte compartment) for predicting 90-day mortality was 0.869 ± 0.078 (*p* = 0.004, 95% CI 0.716–1.000), and the best cutoff was 90%. At this cutoff, cMo frequency showed a sensitivity of 83.3%, specificity of 75.8%, positive predictive value of 38.5%, and negative predictive value of 96.2% for 90 day-mortality. Figure 5a exhibits the Kaplan–Meier curve for 90-day mortality according to cMo frequency at a cutoff of 90%. The Kaplan–Meier survival probability was 96.2% in subjects with cMo frequencies < 90.0% and 61.5% in those with values > 90.0% (*p* = 0.003).

The AUROC of iMo percentage (within the monocyte compartment) for estimating 90-day mortality was 0.944 ± 0.037 (*p* = 0.001, 95% CI 0.872–1.000), and the best cutoff was 5.7%. At this cutoff, iMo frequency showed a sensitivity of 100%, specificity of 87.9%, positive predictive value of 60.0%, and negative predictive value of 100%. The Kaplan–Meier curve for 90-day mortality based on iMo frequency at a cutoff of 5.7% is depicted in Fig. 5b. The Kaplan–Meier survival probability was 100% in subjects with iMo frequencies > 5.7% and 60% in those with values ≤ 5.7% (*p* < 0.001).

The AUROC of TiMas percentage (within the monocyte compartment) for predicting 90-day mortality was 0.874 ± 0.078 (*p* = 0.004, 95% CI 0.721–1.000). At the best cutoff (8.6%), TiMas frequency showed a sensitivity of 83.3%, specificity of 81.8%, positive predictive value of 45.5%, and negative predictive value of 96.4%. Figure 5c shows the Kaplan–Meier curve for 90-day mortality on the basis of TiMas frequency at a cutoff of 8.6%. The survival probability was 96.4% in subjects with TiMas frequencies > 8.6% and 54.5% in those with values < 8.6% (*p* = 0.001).

The AUROC of CD62L^+^ monocytes and pDC/cDC ratio did not show significant results.

## Discussion

In patients with inflammatory liver disease, monocytes are recruited from the blood and then differentiate into distinct functional subsets of macrophages and DCs that regulate inflammation, fibrogenesis, and resolution^33^. In these cases, the phenotype of circulating monocytes may be a valuable indicator of hepatic recruitment and systemic inflammatory state^31^. Previous studies observed an increase in circulating and hepatic CD16^+^ monocytes in cirrhotic patients; these cells are believed to play a role in the perpetuation of inflammatory and profibrogenic signals during liver disease progression^18,31,34^. In this study, we observed an increase of circulating iMo (CD14^+^ CD16^+^) in cirrhotic patients compared with healthy CTR individuals. In addition, we detected alterations in the proportions of circulating monocytes, particularly in patients with more advanced liver disease. These findings suggest intensification of monocyte recruitment from bone marrow to peripheral blood, which results in increased circulating cMo frequency. Depending on the stimuli received, cMo differentiate into iMo, although it is not yet known whether differentiation occurs in circulation or in the inflamed tissue. iMo cells differentiate into ncMo, and, after acting on the tissue, return to peripheral blood^3^. We observed that circulating ncMo levels were reduced in AD patients in comparison with CTR.

CD62L, an adhesion molecule belonging to the selectin family, is expressed by cells for endothelial adherence and tissue migration^35^. Cirrhotic patients had higher percentages of monocytes expressing CD62L than CTR, as also observed in a previous study^31^. This finding suggests intensification of recruitment and migration of monocytes from peripheral blood to inflammatory sites.

HLA-DR is a class II human leukocyte antigen typically expressed in antigen-presenting cells. In this study, HLA-DR expression on cMo was reduced. Such a reduction has been observed in cirrhotic patients^31,36,37^, and is a well-known characteristic of ACLF and septic shock^38^. Furthermore, reduced HLA-DR expression, combined with enhanced IL-6 and IL-10 and decreased IL-1, TNF-a, and nitric oxide synthase, is typical of immune paralysis^16^. The results of the present study indicate that a high TiMas/cMo HLA-DR ratio occurs because of a reduction in HLA-DR expression on cMo and not because of increased expression on iMo or ncMo, which did not differ significantly between groups.

As previously mentioned, when compared with CTR, cirrhotic patients showed high iMo percentages. However, in comparing between cirrhotic patients, we found that those with more advanced disease showed higher cMo and lower iMo and ncMo percentages. Moreover, we found that lower frequencies of iMo and TiMas and higher frequencies of cMo within the monocyte compartment (cutoffs of ≤ 5.7%, < 8.6%, and ≥ 90%, respectively) were associated with mortality. Monocytes are crucial mediators of host defense, and infections are responsible for much of the morbidity and mortality in patients with AD of cirrhosis. This could indicate that the occurrence of CAID in the last stages of liver disease, when the immune system collapses, is marked by the disappearance of TiMas in peripheral blood. These unprecedented findings show that circulating monocyte subsets could represent useful biomarkers in clinical practice.

The current paradigm suggests that pDCs and cDCs have a common myeloid progenitor in the bone marrow, known as monocyte-DC progenitor (MDP), which can differentiate into DC-committed progenitors, giving rise to cDCs and pDCs^11,39^. DCs play a key role in the induction of innate and adaptive immune responses against specific antigens; however, its role in the initiation and progression of liver diseases is poorly elucidated. DCs are thought to promote resolution rather than progression of fibrosis^40^.

CAID is characterized by impairment of phagocytosis and antigen presentation, which might be associated with reduced frequencies or impaired function of circulating DCs^14^. Alterations in DCs, such as numerical reduction, impaired production of inflammatory cytokines, and increased production of immunosuppressive IL-10, have been described in HCV infection^21,22^. Impaired maturation and impaired expression of specific immune checkpoints and TLR molecules were detected in circulating DCs of patients with chronic HBV infection^23,41^. Ouaguia and colleagues found that circulating DCs are reduced in frequency and absolute numbers in patients chronically infected with HBV. The authors suggested the occurrence of a specific mechanism for active recruitment of circulating pDCs to HBV-infected liver^23^.

In the current study, we observed a profound reduction in both absolute and relative numbers of circulating DCs in cirrhotic patients, especially in those with signs of advanced liver disease. The proportion of pDCs within the DC compartment was higher in cirrhotic patients, mainly in the AD group, resulting in an augmented pDC/cDC ratio. Despite the proinflammatory role of pDCs (exerted by secretion of type I IFN), these cells were associated with induction of immune tolerance and immunosuppression. In transplant patients, high pDC/cDC ratios were associated with lower rejection rates^42–44^.

The cytokine profile observed in this study—elevated plasma levels of IL-6 and IL-10, particularly in AD patients, and elevated plasma levels of IL-17A, particularly in SC patients—corroborates the results of a previous study carried out by our research group, which analyzed a cohort of 118 SC patients, 130 patients hospitalized for AD cirrhosis, and 30 healthy controls^45^. It has been reported that high levels of Th2-related cytokines, such as IL-10, are associated with chronic HCV infections^46^. In vitro tests showed that cMo isolated from peripheral blood differentiate into iMo after stimulation with TGF-β and IL-10^47^, indicating that IL-10 could be related to the increased numbers of circulating iMo in cirrhotic patients compared with healthy individuals. In accordance with a previous study^45^, here, IL-6 levels were found to be elevated in cirrhotic patients, mainly in those with advanced liver disease. IL-6 and IL-4 are associated with Th cell differentiation into Th2 and deeply involved in monocyte activation and chemotaxis toward the inflamed region^46^. IL-17A is a highly versatile proinflammatory cytokine produced by Th17 cells. The significance of circulating IL-17A has not been completely elucidated, particularly in the case of patients with hepatic diseases. A possible explanation for the low IL-17A levels in AD patients as compared with SC patients is the inhibitory effect of high IL-10 levels on Th17 cells^48^.

Overall, our findings indicate the presence of systemic effects induced by liver cirrhosis that influence the immune–hematopoietic system. CAID profile varied according to disease severity, as evidenced by phenotypic differences between cirrhotic patients at different stages of liver disease. The results revealed alterations in circulating monocyte levels and their differentiation to TiMas, as well as an important reduction of circulating DCs and increased pDC/cDC ratio in cirrhotic patients. A greater understanding of the immunological aspects of liver cirrhosis may contribute to the development of improved therapeutic approaches and facilitate detection of patients that could benefit most from immunotherapy.

## Methods

### Patients and controls

This prospective cohort study included consecutive adult cirrhotic patients hospitalized for acute decompensation (AD) at the University Hospital of the Federal University of Santa Catarina, Brazil, between March 2018 and December 2018. In addition to hospitalized patients, 29 adult cirrhotic patients attending the outpatient clinic at the same hospital were included as the stable cirrhosis (SC) group. Cirrhosis diagnosis was established histologically or by a combination of clinical, imaging, and laboratory findings in patients with evidence of portal hypertension.

Patients with hepatocellular carcinoma, receiving immunosuppressive therapy, seropositive for human immunodeficiency virus (HIV), with active Epstein–Barr virus or cytomegalovirus infection, with a history of neoplasm (except skin cancer) or cirrhosis of autoimmune etiology were excluded from the study. A questionnaire was used to collect information not available from medical records. Laboratory results were obtained for SC and AD cohorts.

Thirty age- and gender-matched healthy subjects undergoing routine health examination were selected as the control (CTR) group, using AD group as reference. Healthy individuals were interviewed using a questionnaire. Exclusion criteria included history of hepatic disease, history of neoplasm (except skin cancer), infections in the previous seven days, heavy alcohol consumption, diabetes mellitus, autoimmune disease, chronic inflammatory disease, and use of immunosuppressive therapy.

Written informed consent was obtained from all participants. The study was approved by the Ethics Committee of the Federal University of Santa Catarina (CEPSH UFSC protocol no. 1822/2011).

Correlations between laboratory and sociodemographic variables were investigated. Child–Pugh^26^ and Model for End-Stage Liver Disease (MELD) scores^27,28^ were used to assess the severity of hepatic disease. Acute-on-chronic liver failure (ACLF) was defined as proposed by the EASL-CLIF Consortium^29^.

Peripheral blood was drawn into 4 mL vacutainers (Vacuette, Greiner Bio-One, Austria) containing ethylenediaminetetraacetic acid (EDTA) for anticoagulation. Samples were obtained at routine office visits for SC patients and on the first day of hospitalization for the AD cohort. Blood from all individuals was processed within 24 h of collection for flow cytometric analysis and plasma separation. Plasma was stored at − 80 °C until cytokine analysis.

### Immunophenotyping of monocytes and DCs by flow cytometry

The staining protocol was lyse-wash. The antibodies used for phenotyping included HLA-DR (PacB, L243), CD45 (PacO HI30), CD123 (PE, 7G3), CD10 (PECy7, HI10a), CD14 (APC, MφP9), CD3 (APC-H7, SK7), and CD20 (APC-H7, 2H7) from BD Biosciences (San Jose, CA, USA); CD16 (FITC, G38) from Beckman Coulter (Brea, CA, USA); CD62L (FITC, LT-TD180), CD4 (PerCP, MEM-241), and CD11c (PerCP, BU15) from Exbio (Prague, Vestec, Czech Republic); and CD19 (APC-H7, HIB19) from BioLegend (San Diego, CA, USA). Technical information on reagents can be found in Table S1 (Supplementary material).

Peripheral blood-EDTA samples (300 µL) were stained with an eight-color panel previously designed and tested by our group to identify monocyte and DC subsets. CD62L expression was analyzed by using a different panel, which also contained antibodies for monocyte identification (HLA-DR, CD4, and CD45). Detailed information on staining procedures and acquisition parameters can be found in our previous study^30^.

**Figure 6:**
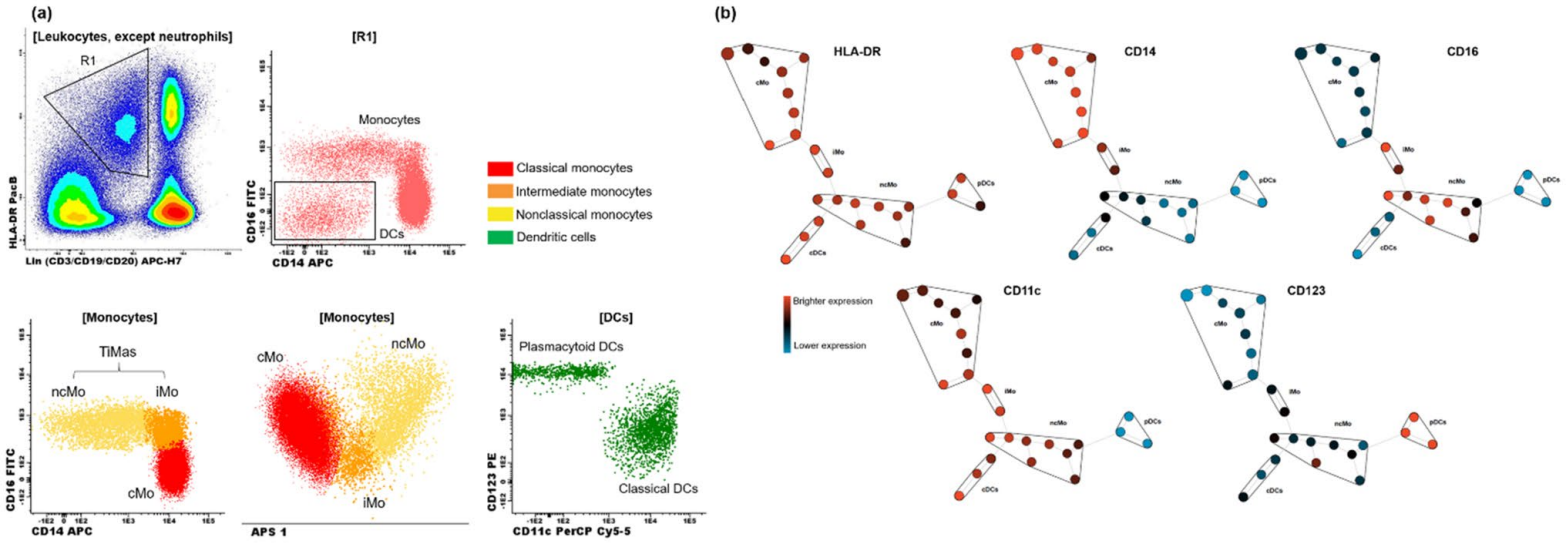
Panel (**a**) Gating strategy of circulating monocyte and dendritic cell (DC) subsets by multiparameter flow cytometry. Cells that were negative for CD3, CD19 and CD20 (lineage negative) and HLA-DR^+^ were gated (R1). CD14 and CD16 negative cells (DCs) were then separated from monocytes. Classification of classical (CD14^+^ CD16^neg^), intermediate (CD14^+^ CD16^+^) and nonclassical monocytes (CD14^lo^ CD16^+^). DCs were classified according to CD11c and CD123 expression in classical (cDC) (CD11c^+^ CD123^lo^) and plasmacytoid DCs (pDC) (CD123^++^ CD11c^neg^). Panel (**b**) Monocyte and DC clusters identified by SPADE. Cellular phenotypes were assigned to the SPADE tree, based on node expression and location. *cDC* classical dendritic cell, *cMo* Classical monocytes, *DCs* Dendritic cells, *iMo* Intermediate monocytes, *ncMo* Nonclassical monocytes, *pDC* Plasmacytoid dendritic cell, *TiMas* Tissular macrophages (iMo + ncMo).

Data acquisition was performed using a three-laser FACSCanto II flow cytometer equipped with FACSDiva software version 6 (BD Biosciences, USA). For homogeneous cell analysis with good sensitivity, 500,000 to 1,000,000 gated CD45^+^ events were recorded for each individual. Data were analyzed using Infinicyt version 1.7.0 (Cytognos, Spain) and the web-based application Cytobank 7.3.0 (Beckman Coulter, USA) (Fig. 6). The gating strategy was adapted from Cardoso and Santos-Silva^30^ and previous studies^20,31, 32^. Cutoff limits were defined based on fluorescence-minus-one (FMO), negative, and positive controls. Automatic gates, applied using the analysis strategy tool, were supervised by two operators during analysis. Absolute numbers were calculated by a dual-platform flow cytometry method using a XE 2100 blood cell analyzer (Sysmex, Japan).

### HLA-DR and CD62L expression on monocytes

Analysis of HLA-DR expression on monocytes was performed by determining the mean fluorescence intensity (MFI) of this marker on cMo, iMo, ncMo, and TiMas. MFI values were then compared between groups. The MFI ratio of TiMas HLA-DR to cMo HLA-DR (hereafter referred to as TiMas/cMo HLA-DR ratio) was calculated to reduce the variability associated with antibody batches and equipment conditions. The ratio is expected to be positive, as ncMo and iMo have greater HLA-DR expression than cMo. Because CD62L expression is bimodal, we chose to report the values of monocytes expressing this molecule as a percentage. HLA-DR and CD62L expression was also analyzed by the tool heatmap of the web-based application Cytobank 7.3.0 (Beckman Coulter, USA).

### Quantification of cytokine plasma levels

Quantitative determination of interleukin (IL)-2, IL-4, IL-6, IL-10, tumor necrosis factor-α (TNF-α), interferon-γ (IFN-γ), and IL-17A in plasma was performed using a cytometric bead array (CBA) human Th1/Th2/Th17 kit (BD Biosciences, USA). The fluorescence produced by CBA beads was measured on a FACSCanto II flow cytometer (BD Biosciences, USA) and analyzed using FCAP Array version 3.0 (BD Biosciences, USA).

### Statistical analysis

The normality of data distribution was assessed using Shapiro–Wilk test. Continuous variables were compared using Student’s *t*-test or analysis of variance (ANOVA) with Post-hoc Bonferroni in the case of normal distribution and Mann–Whitney U or Kruskal–Wallis tests in the remaining cases. Categorical variables were evaluated with the chi-square test. Correlations between numerical variables were evaluated using Spearman’s correlation coefficient. Univariate and multivariate Cox regression analyses were used to evaluate the association between variables of interest and survival. The best cutoffs of monocytes for predicting mortality were chosen on the basis of the area under receiver operating characteristic (AUROC) curves. Survival curves were calculated by the Kaplan–Meier method, and survival differences between groups were compared using a log-rank test. *P*-values of equal or less than 0.05 were considered statistically significant. Statistical analysis were performed using SPSS software version 17.0 (SPSS, Chicago, IL, USA).

### Ethics approval

This study was conducted in accordance with the 1964 Helsinki declaration and Brazilian National Health Council Resolution No. 196/1996. Experimental protocols were approved by the Human Research Ethics Committee of the Federal University of Santa Catarina, Brazil.

### Informed consent

Informed consent was obtained from all individual participants included in this study.

## Supplementary Information

Supplementary Information

## Abbreviations

DCs: Dendritic cells
cMo: Classical monocytes
iMo: Intermediate monocytes
ncMo: Nonclassical monocytes
TiMas: Tissular macrophages
Th: T helper
Tc: Cytotoxic T cells
cDCs: Classical DCs
pDCs: Plasmacytoid DCs
TLRs: Toll-like receptors
CAID: Cirrhosis-associated immune dysfunction
HCV: Hepatitis C virus
HBV: Hepatitis B virus
HCC: Hepatocellular carcinoma
AD: Acute decompensation
SC: Stable cirrhosis
HIV: Human immunodeficiency virus
CTR: Control group
MELD: Model for end-stage liver disease
ACLF: Acute-on-chronic liver failure
PB: Peripheral blood
EDTA: Ethylenediaminetetraacetic acid
MFI: Mean fluorescence intensity
IL: Interleukin
CBA: Cytometric bead array
ROC: Receiver operating characteristics
NASH: Nonalcoholic steatohepatitis
SBP: Spontaneous bacterial peritonitis
HE: Hepatic encephalopathy
CC: Compensated cirrhosis
SCD: Stable cirrhosis that had already developed decompensated cirrhosis
HLA: Human leucocyte antigen
MDP: Monocyte-DC progenitor
