# Supplementary material for: Patterns of dendritic cell and monocyte subsets are associated with disease severity and mortality in liver cirrhosis patients
Source: Sci Rep. 2021 Mar 15;11:5923. doi: 10.1038/s41598-021-85148-y (PMC7960697; doi:10.1038/s41598-021-85148-y)
Supplement: Supplementary file 1 — Supplementary Information [file 41598_2021_85148_MOESM1_ESM.pdf]

Supplementary material

Title page

Patterns of Dendritic Cell and Monocyte Subsets are Associated with Disease Severity and Mortality in Liver Cirrhosis Patients

Chandra Chiappin Cardoso[1,2], Camila Matiollo[3], Carolina Hilgert Jacobsen Pereira[2], Janaina Sant´ana Fonseca[4], Helder Emmanuel Leite Alves[4], Otavio Marcos da Silva[4], Vivian de Souza Menegassi[4], Claudia Regina dos Santos[2,5], Ana Carolina Rabello de Moraes[2,5], Leonardo de Lucca Schiavon[3,4], Maria Claudia Santos-Silva[1,2,5]

[1] Division of Clinical Analysis, Flow Cytometry Service, University Hospital of the Federal University of Santa Catarina, Florianópolis, SC, Brazil
[2] Postgraduate Program in Pharmacy of the Federal University of Santa Catarina, Florianópolis, SC, Brazil
[3] Postgraduate Program in Medical Sciences of the Federal University of Santa Catarina, Florianópolis, SC, Brazil
[4] Division of Gastroenterology, Federal University of Santa Catarina, Florianópolis, SC, Brazil
[5] Clinical Analysis Department, Health Sciences Center, Federal University of Santa Catarina, Florianópolis, SC, Brazil

Article title: Patterns of Dendritic Cell and Monocyte Subsets are Associated with Disease Severity and Mortality in Liver Cirrhosis Patients
Authors: Cardoso CC, et al.
Federal University of Santa Catarina (UFSC), Brazil
E-mail: chandraccardoso@gmail.com

Table S1 Technical information on reagents.

Marker	Fluorochrome	Clone	Source	Catalogue Number	µL/test
CD3	APCH7	SK7	BD Bioscience	557832	5
CD4	PerCP Cy5-5	MEM-241	Exbio	PC-359-T100	1
CD10	PECy7	HI10A	BD Bioscience	341092	3
CD11c	PerCP Cy5-5	BU15	Exbio	T9-529-T100	1
CD14	APC	MφP9	BD Bioscience	345787	2
CD16	FITC	3G8	Beckman Coulter	IM0814U	7
CD19	APCH7	HIB19	Biolegend	302218	1
CD20	APCH7	2H7	BD Bioscience	560853	1
CD45	PacO	HI30	BD Bioscience	560777	1
CD62L	FITC	LT-TD180	Exbio	1F-449-T100	15
CD123	PE	7G3	BD Bioscience	554529	3
HLA-DR	PacB	L243	BD Bioscience	642276	2

APC: allophycocyanin; APCH7: allophycocyanin H7; FITC: fluorescein isothiocyanate; PacB: pacific blue/V450; PacO: pacific Orange/V500; PE: phycoerythrin; PECy7: phycoerythrin Cy7; PerCP Cy5-5: peridinin chlorophyll protein; Ig: immunoglobulin.

Table S2 Frequencies of monocytes and DCs and levels of cytokines in healthy controls, stable cirrhosis and in acute decompensation of cirrhosis.

	Healthy controls (n=30)		Stable cirrhosis (n=29)		Acute decompensation of cirrhosis (n=39)	
**Cell subset**	**Mean ± SD**	**Median (range)**	**Mean ± SD**	**Median (range)**	**Mean ± SD**	**Median (range)**
**Monocytes %**	6.90 ± 1.55	6.94 (3.76 – 9.69)	7.31 ± 2.73	7.12 (3.54 – 14.90)	9.16 ± 3.62	8.90 (1.92 – 16.70)
**Monocytes mm^3**	457 ± 128	442 (281 – 740)	427 ± 209	388 (138 – 966)	525 ± 333	450 (53 – 1,364)
**cMo %**	5.85 ± 1.45	5.75 (3.16 – 8.67)	5.98 ± 2.33	5.52 (2.27 – 13.97)	7.85 ± 3.11	7.57 (1.63 – 14.06)
**cMo %†**	84.22 ± 4.60	84.63 (75.59 – 92.42)	81.72 ± 8.14	82.05 (64.09 – 93.93)	85.82 ± 7.86	87.98 (67.66 – 99.63)
**cMo mm^3**	388 ± 120	363 (238 – 683)	346 ± 160	310 (109 – 657)	451 ± 288	392 (47 – 1.329)
**iMo %**	0.40 ± 0.10	0.40 (0.22 – 0.68)	0.81 ± 0.76	0.53 (0.21 – 3.92)	0.95 ± 0.85	0.63 (0.24 – 3.63)
**iMo %†**	6.01 ± 1.55	5.56 (3.61 – 9.22)	10.62 ± 6.64	8.41 (3.20 – 29.12)	9.88 ± 6.11	7.55 (0.29 – 23.45)
**iMo mm^3**	27 ± 8	26 (14 – 41)	50 ± 54	40 (8 – 281)	54 ± 64	33 (4 – 297)
**NcMo %**	0.65 ± 0.29	0.60 (0.19 – 1.25)	0.52 ± 0.33	0.46 (0.08 – 1.66)	0.36 ± 0.29	0.25 (0.01 – 1.07)
**NcMo %†**	9.77 ± 4.02	9.23 (2.07 – 17.47)	7.66 ± 4.77	6.89 (1.41 – 20.97)	4.29 ± 3.23	3.32 (0.08 – 12.27)
**NcMo mm^3**	43 ± 19	38 (16 – 80)	31 ± 24	26 (4 – 121)	19 ± 17	13 (1 – 85)
**TiMas %**	1.06 ± 0.34	1.00 (0.46 – 1.86)	1.33 ± 0.86	1.15 (0.30 – 4.31)	1.31 ± 1.00	0.97 (0.27 – 4.01)
**TiMas %†**	15.78 ± 4.60	15.37 (7.58 – 24.41)	18.28 ± 8.14	17.95 (6.07 – 35.91)	14.26 ± 7.72	12.02 (2.53 – 32.34)
**TiMas mm^3**	69 ± 23	64 (35 – 113)	81 ± 66	65 (12 – 309)	74 ± 74	51 (6 – 328)
**HLA-DR cMo**	9,911 ± 3,956	9,852 (2,701 – 18,734)	8,838 ± 3,888	8,329 (1,442 – 19,108)	6,738 ± 4,014	6,494 (1,548 – 20,879)
**HLA-DR TiMas/cMo (ratio)**	1.81 ± 0.46	1.67 (1.07 – 2.78)	2.16 ± 0.67	2.04 (1.17 – 3.98)	2.89 ± 1.23	2.67 (1.21 – 6.95)
**CD62L %**	65.49 ± 14.28	70.04 (33.45 – 86.55)	76.48 ± 11.60	78.66 (37.79 – 92.98)	76.71 ± 17.78	84.20 (28.49 – 97.40)
**DC %**	0.33 ± 0.10	0.31 (0.16 – 0.62)	0.25 ± 0.10	0.26 (0.06 – 0.44)	0.18 ± 0.13	0.15 (0.00 – 0.62)
**DC mm^3**	22 ± 7	19 (10 – 39)	14 ± 7	13 (6 – 26)	9 ± 6	7 (0 – 24)
**cDC %**	0.23 ± 0.09	0.21 (0.11 – 0.56)	0.16 ± 0.08	0.16 (0.03 – 0.34)	0.10 ± 0.07	0.08 (0.00 – 0.34)
**cDC %‡**	68.65 ± 10.29	70.37 (45.42 – 90.00)	63.53 ± 13.36	65.29 (31.79 – 80.46)	57.86 ± 14.30	54.89 (23.93 – 84.43)
**cDC mm^3**	15 ± 7	13 (7 – 35)	9 ± 5	8 (2 – 18)	5 ± 4	4 (0 – 16)
**pDC %**	0.10 ± 0.03	0.09 (0.04 – 0.18)	0.09 ± 0.04	0.09 (0.03 – 0.22)	0.08 ± 0.06	0.06 (0.00 – 0.28)
**pDC %‡**	31.36 ± 10.29	29.63 (10.00 – 54.58)	36.47 ± 13.36	34.71 (19.54 – 68.21)	42.12 ± 14.31	45.11 (15.57 – 76.07)
**pDC mm^3**	6 ± 2	6 (3 – 11)	5 ± 2	4 (1 – 10)	4 ± 3	3 (0 – 15)
**pDC/cDC**	0.49 ± 0.25	0.42 (0.11 – 1.20)	0.67 ± 0.47	0.53 (0.24 – 2.15)	0.85 ± 0.54	0.82 (0.18 – 3.18)
**IL-17A**	0.12 ± 0.50	0.00 (0.00 – 2.60)	3.25 ± 4.87	0.00 (0.00 – 17.69)	0.00 ± 0.00	0.00 (0.00 – 0.00)
**IL-10**	0.02 ± 0.06	0.00 (0.00 – 0.35)	0.97 ± 1.79	0.35 (0.00 – 7.46)	1.47 ± 3.87	0.74 (0.00 – 24.25)
**IL-6**	0.53 ± 0.64	0.35 (0.00 – 3.04)	2.75 ± 3.34	1.60 (0.34 – 14.29)	120.61 ± 340.63	17.95 (4.06 – 1,978.06)

† The percentage of monocytes subsets referred to as total monocytes. ‡ The percentage of cDC and pDC referred to as total DCs. SD = standard deviation; cMo = classical monocytes; iMo = intermediate monocytes ; ncMo = nonclassical monocytes; TiMas = tissular macrophages (iMo + ncMo); DCs = dendritic cells; cDC = classical dendritic cells; pDC = plasmacytoid dendritic cells; IL = interleukine.

Table S3 Spearman's correlation coefficient between monocytes, DCs and cytokines with MELD and Child-Pugh scores in liver cirrhosis patients.

**Variables**	**MELD**		**Child-Pugh**	
	$\mathbf{r_s}$	***p***	$\mathbf{r_s}$	***p***
**Monocytes %**	0.268	0.027	0.313	0.009
**cMo %**	0.302	0.012	0.376	0.002
**cMo %†**	0.207	0.091	0.321	0.008
**cMo mm^3**	0.148	0.229	0.249	0.040
**NcMo%**	-0.257	0,035	-0.324	0.007
**NcMo % †**	-0.355	0.003	-0.425	<0.001
**NcMo mm^3**	-0.286	0.018	-0.320	0.008
**TiMas % †**	-0.206	0.092	-0.320	0.008
**HLA-DR cMo**	-0.329	0.006	-0.298	0.014
**HLA-DR TiMas/cMo**	0.455	<0.001	0.290	0.017
**CD62L %**	0.256	0.035	0.163	0.183
**DCs %**	-0.329	0.006	-0.321	0.008
**DCs mm^3**	-0.440	<0.001	-0.353	0.003
**cDC %**	-0.414	<0.001	-0.447	<0.001
**cDC % ‡**	-0.269	0.026	-0.429	<0.001
**cDC mm^3**	-0.494	<0.001	-0.450	<0.001
**pDC % ‡**	0.269	0.026	0.429	<0.001
**pDC mm3**	-0.251	0.039	-0.124	0.313
**pDC/cDC**	0.269	0.026	0.429	<0.001
**IL-17A**	-0.438	<0.001	-0.529	<0.001
**IL-10**	0.210	0.085	0.250	0.040
**IL-6**	0.672	<0.001	0.761	<0.001

† The percentage of monocytes subsets referred to as total monocytes. ‡ The percentage of cDC and pDC referred to as total DCs. The correlations between numerical variables were evaluated using Spearman's correlation coefficient (r_s). *P* values indicate whether there was a significant correlation. cDC = classical dendritic cell; cMo = classical monocytes; DCs = dendritic cells; IL = interleukine; ncMo = nonclassical monocytes; pDC = plasmacytoid dendritic cell; TiMas = tissular macrophages.

Table S4 Characteristic of AD patients with and without ACLF.

Variable	AD without ACLF (n = 31)	AD with ACLF (n = 8)	*p*
**Age (years), mean ± SD**	55.48 ± 12.06	59.50 ± 9.56	0.389
**Male gender, n (%)**	10 (32.3)	2 (25.0)	0.527
**Laboratorial parameters**			
**Leukocytes/mm^3, median (range)**	5,060 (1,010 – 14,510)	8,035 (4,050 – 10,940)	0.092
**Monocytes/mm^3, median (range)**	332.8 (47.3 – 1,329.1)	529.7 (293.2 – 1,052.6)	0.085
**Monocytes (%), mean ± SD**	9.13 ± 3.74	9.31 ± 3.38	0.902
**Monocytes subsets †**			
**cMo, median (range)**	85.9 (67.7 – 99.6)	91.2 (74.1 – 93.9)	0.173
**iMo, median (range)**	8.30 (0.29 – 23.15)	5.45 (4.10 – 23.45)	0.048
**ncMo, mean ± SD**	4.45 ± 3.40	3.71 ± 2.56	0.570
**TiMas, median (range)**	14.08 (2.53 – 32.34)	8.81 (6.13 – 25.90)	0.173
**DCs/mm^3, median (range)**	8.99 (0.00 – 23.90)	4.71 (0.83 – 21.28)	0.173
**DCs (%), median (range)**	0.19 (0.00 – 0.62)	0.07 (0.01 – 0.27)	0.015
**DCs subsets ‡**			
**cDC, mean ± SD**	58.75 ± 12.46	54.39 ± 20.69	0.449
**pDC, mean ± SD**	41.22 ± 12.47	45.61 ± 20.69	0.447
**pDC/cDC ratio, mean ± SD**	0.77 ± 0.35	1.13 ± 0.97	0.332
**Platelets/mm^3, median (range)**	68,000 (18,000 – 245,000)	72,000 (39,000 – 259,000)	0.645
**Sodium (mEq/L), mean ± SD**	136.84 ± 4.86	135.87 ± 6.96	0.650
**Creatinine (mg/dL), median (range)**	1.09 (0.57 – 1.70)	2.22 (0.55 – 6.19)	0.003
**INR, median (range)**	1.44 (1.04 – 2.16)	1.47 (1.17 – 2.58)	0.720
**Albumin (g/dL), mean ± SD**	2.92 ± 0.57	2.72 ± 1.05	0.632
**CRP (mg/L), median (range)**	14.90 (3.20 – 172.90)	48.85 (12.70 – 162.70)	0.023
**Total bilirubin (mg/dL), median (range)**	2.10 (0.20 – 8.60)	1.70 (0.60 – 8.90)	0.597
**Lactate (mmol/L), median (range)**	1.55 (0.80 – 4.90)	2.00 (1.50 – 2.60)	0.183
**AST (IU/L), median (range)**	46 (20 – 260)	37 (15 – 70)	0.173
**ALT (IU/L), median (range)**	36 (8 – 165)	22 (14 – 32)	0.012
**GGT (IU/L), median (range)**	114 (34 – 536)	94 (18 – 261)	0.346
**IL-10 (pg/mL), median (range)**	0.41 (0.00 – 24.25)	1.43 (0.51 – 4.09)	0.005
**Complication at evaluation, n (%)**			
**Ascites**	19 (61.3)	7 (87.5)	0.229
**Hepatic encephalopathy**	12 (38.7)	7 (87.5)	0.020
**Gastrointestinal bleeding**	15 (48.4)	2 (25.0)	0.426
**Bacterial infection**	2 (6.5)	2 (25.0)	0.180
**Child–Pugh, median (range)**	9 (6 – 13)	10 (7 – 14)	0.173
**Child–Pugh A, n (%)**	4 (12.9)	0 (0.0)	0.563
**Child–Pugh B, n (%)**	16 (51.6)	3 (37.5)	0.695
**Child–Pugh C, n (%)**	11 (35.5)	5 (62.5)	0.235
**MELD, median (range)**	14 (8 – 22)	21 (14 – 29)	0.007
**MELD < 9, n (%)**	3 (9.7)	0 (0.0)	0.492
**MELD 10 – 19, n (%)**	24 (77.4)	3 (37.5)	0.079
**MELD 20 – 29, n (%)**	4 (12.9)	5 (62.5)	0.009

† The percentage of monocytes subsets referred to as total monocytes. ‡ The percentage of cDC and pDC referred to as total DCs. AD = acute decompensation of cirrhosis; SD = standard deviation; Mo = monocytes, cMo = classical monocytes; iMo = intermediate monocytes ; ncMo = nonclassical monocytes; TiMas = tissular macrophages (iMo + ncMo); DCs = dendritic cells; cDC = classical dendritic cells; pDC = plasmacytoid dendritic cells; AST = aspartate aminotransferase; ALT = alanine aminotransferase; CRP = C-reactive protein; GGT = gamma-glutamyl transferase; IL = interleukine; INR = international normalized ratio; MELD = model for end–stage liver disease.

Table S5 Univariate Cox-regression analysis of factors associated with 90-days mortality among patients hospitalized for acute decompensation of cirrhosis.

Parameter	Survivors (n = 33)	Non-survivors (n = 6)	Univariate analysis HR (95% CI)	*p*
**Age (years), mean ± SD**	54.79 ± 10.96	64.67 ± 12.40	1.086 (0.995 – 1.186)	0.064
**Male Gender, n (%)**	24 (72.7)	3 (50.0)	2.426 (0.489 – 12.022)	0.278
**Active alcoholism, n (%)**	6 (18.6)	1 (16.7)	1.170 (0.476 – 2.875)	0.732
**Complication at admission, n (%)**				
**Ascites**	20 (60.6)	6 (100.0)	4.098 (0.390 – 33.161)	0.296
**Hepatic encephalopathy**	15 (45.5)	4 (66.7)	2.260 (0.414 – 12.351)	0.347
**Gastrointestinal bleeding**	15 (45.5)	2 (33.3)	0.640 (0.117 – 3.493)	0.606
**Bacterial infection**	1 (3.0)	3 (50.0)	12.986 (2.515 – 67.038)	0.002
**Laboratory data**				
**Leukocyte/mm^3, median (range)**	5,060 (1,010 – 14,510)	8,240 (3,100 – 11,340)	1.189 (0.953 – 1.483)	0.124
**Monocytes (%)**	8.95 ± 3.62	10.30 ± 3.78	1.002 (1.000 – 1.004)	0.052
**cMo †**	84.51 ± 7.70	93.06 ± 4.07	1.254 (1.065 – 1.478)	0.007
**iMo †**	10.89 ± 6.11	4.33 ± 1.19	0.683 (0.526 – 0.885)	0.004
**ncMo †**	4.60 ± 3.19	2.62 ± 3.13	0.747 (0.494 – 1.130)	0.167
**TiMas †**	15.59 ± 7.52	6.95 ± 4.07	0.698 (0.537 – 0.906)	0.007
**CD62L$^+$**	84.20 (28.97 – 97.40)	61.81 (28.49 – 96.00)	0.959 (0.926 – 0.994)	0.021
**Dendritic cells (%)**	0.17 (0.00 – 0.62)	0.08 (0.03 – 0.29)	0.000 (0.000 – 7.566)	0.120
**cDC ‡**	59.72 ± 13.25	47.64 ± 16.84	0.929 (0.864 – 1.000)	0.049
**pDC ‡**	40.26 ± 13.25	52.36 ± 16.84	1.076 (1.000 – 1.158)	0.049
**pDC/cDC ratio**	0.75 ± 0.36	1.37 ± 0.99	3.842 (1.444 – 10.221)	0.007
**Sodium (mEq/L), mean ± SD**	137.06 ± 4.75	134.33 ± 7.66	0.919 (0.787 – 1.074)	0.288
**Creatinine (mg/dL), median**	1.11 (0.55 – 6.19)	1.28 (0.94 – 2.48)	1.037 (0.483 – 2.224)	0.926
**INR, median**	1.51 ± 0.29	1.74 ± 0.52	5.348 (0.677 – 42.215)	0.112
**Albumin (g/dL), mean ± SD**	2.90 ± 0.65	2.75 ± 0.91	0.743 (0.217 – 2.544)	0.637
**CRP (mg/L), median**	14.90 (3.20 – 172.90)	88.30 (21.20 – 158.00)	1.016 (1.004 – 1.028)	0.011
**Total bilirubin (mg/dL), median**	2.10 (0.20 – 8.60)	1.85 (0.60 – 8.90)	1.240 (0.911 – 1.686)	0.171
**IL-6 (pg/mL), median**	13.39 (4.06 – 415.13)	264.37 (48.06 –	1.015 (1.005 – 1.025)	0.004
**ACLF, n (%)**	5 (15.2)	3 (50.0)	4.275 (0.858 – 21.197)	0.076
**MELD score, median**	16 (8 – 25)	17 (11 – 29)	1.114 (0.945 – 1.312)	0.198
**Child-Pugh C, n (%)**	11 (33.3)	5 (83.3)	8.635 (1.006 – 74.092)	0.049

† The percentage of monocytes subsets referred to as total monocytes. ‡ The percentage of cDC and pDC referred to as total DCs SD = standard deviation; WBC = white blood cells; Mo = monocytes, cMo = classical monocytes; iMo = intermediate monocytes; ncMo = nonclassical monocytes; TiMas = tissular macrophagues (iMo + ncMo); DCs = dendritic cells; cDC = classical dendritic cell; pDC = plasmacytoid dendritic cell; AST = aspartate aminotransferase; ALT = alanine aminotransferase; CRP = C-reactive protein; GGT = gamma-glutamyl transferase; INR = international normalized ratio; MELD = model for end–stage liver disease.
